# IT WORKS FOR US: BRIDGING THE GAP BETWEEN EDUCATION, PRACTICE, AND POLICY IN GERONTOLOGY AT MIAMI UNIVERSITY

**DOI:** 10.1093/geroni/igad104.1776

**Published:** 2023-12-21

**Authors:** Jennifer Kinney, Katherine Abbott, Sara McLaughlin, Leah Janssen, Robert Applebaum

**Affiliations:** Miami University, Oxford, Ohio, United States; Miami University, Oxford, Ohio, United States; Miami University, Oxford, Ohio, United States; Miami University, Oxford, Ohio, United States; Miami University, Oxford, Ohio, United States

## Abstract

We describe a unique and successful partnership between the Department of Sociology and Gerontology and the Scripps Gerontology Center at Miami University in Oxford, Ohio. This partnership concurrently facilitates research that informs aging policy and gerontological training for students. After a brief description of the partnership, examples of current research that contributes to public policy at the federal, state, and local/provider levels are presented, along with students’ roles in these efforts. Next, we share factors that contribute to our success (e.g., attracting students from diverse backgrounds, using a shared mentorship model) and challenges (e.g., that research and policy move at different paces). We conclude with a look to the future, specifically, the need to engage students in our academic programs, maintain our long-standing relationships, and build new, cross-organizational ties so that future gerontologists will continue our mission of doing work that positively impacts older people.

